# A Multi-Dimensional Vision-Based System for External Thread Defect Detection with Integrated Security Defense

**DOI:** 10.3390/s26103229

**Published:** 2026-05-20

**Authors:** Leqi Li, Gengpei Zhang

**Affiliations:** College of Electronic Information and Electrical Engineering, Yangtze University, Jingzhou 434100, China

**Keywords:** external thread defect detection, point cloud reconstruction, point cloud filtering, data augmentation, security defense

## Abstract

This paper proposes a multi-dimensional vision-based system for external thread defect detection, aiming to overcome the limitations of conventional 2D inspection in geometric characterization. The proposed framework integrates 2D detection and 3D reconstruction to enable both accurate localization and quantitative analysis of defects. Specifically, a YOLOv13-based detector enhanced with data augmentation is employed to detect missing teeth, scratches, and corrosion defects, achieving average precisions of 95.3%, 96.7%, and 79.7%, respectively. To further capture geometric details, a Gaussian Splatting-based reconstruction method is introduced to recover high-fidelity 3D structures from multi-view images. Based on the reconstructed point cloud, dedicated 3D analysis methods are designed to enable defect size estimation with an error of less than 1 mm. Experimental results demonstrate that the proposed system achieves a favorable balance between detection accuracy and geometric measurement capability under complex industrial conditions. In addition, a robustness analysis under image perturbations is conducted to evaluate system reliability.

## 1. Introduction

As one of the most widely used fastening structures in mechanical systems, external threads play a critical role in ensuring structural integrity and operational safety. In practical applications, defects such as missing teeth, scratches, and corrosion may occur during manufacturing and service. These defects, although often small in scale, can significantly affect the load-bearing capacity and reliability of threaded connections [1,2]. Therefore, accurate and reliable defect detection for external threads is of great importance in industrial inspection.

With the rapid development of machine vision and deep learning, 2D image-based defect-detection methods have been widely applied in industrial scenarios. In particular, convolutional neural networks and one-stage detectors, such as the YOLO series, have demonstrated strong performance in detecting surface defects with high efficiency and accuracy [3,4,5,6,7,8,9,10]. However, these methods mainly rely on appearance features extracted from images and lack the ability to capture geometric information, such as depth, volume, and structural deformation. As a result, they are typically limited to identifying the presence of defects while failing to provide a quantitative evaluation of defect geometry.

External threads exhibit complex spatial geometries, including helical structures, high curvature variations, and millimeter-scale depth changes. These characteristics make it difficult to accurately represent defect geometry using only 2D image information, especially under varying viewpoints and lighting conditions. Consequently, relying solely on 2D methods is insufficient for applications that require precise geometric analysis and measurement of defects.

To address these limitations, 3D reconstruction techniques have been introduced to recover geometric information from multi-view observations. Recent advances in image-based reconstruction methods, such as Gaussian Splatting, enable the generation of high-fidelity point clouds using only standard imaging systems, providing a flexible and cost-effective alternative to traditional hardware-based solutions [11,12,13,14,15,16,17,18]. However, applying these techniques to external thread inspection remains challenging due to the complex helical structure, high-frequency geometric variations, and the presence of small-scale defects. Achieving reliable reconstruction and accurate defect quantification under such conditions remains an open problem.

Therefore, a key challenge is how to effectively integrate 2D detection and 3D reconstruction to achieve both reliable defect localization and precise geometric quantification for external threads.

In addition, the robustness of vision-based inspection systems under image perturbations has attracted increasing attention. Image-level disturbances may degrade detection performance, making robustness evaluation an important consideration for practical industrial deployment [19,20,21,22,23,24].

To address the above challenges, this paper proposes a multi-dimensional vision-based external thread defect detection framework that integrates 2D detection and 3D reconstruction. The proposed system first employs a deep-learning-based detector to accurately localize defect regions in images. Based on these results, targeted 3D reconstruction is performed to recover detailed geometric information. Furthermore, dedicated point cloud analysis methods are designed to enable millimeter-level defect quantification.

**Figure 1:**
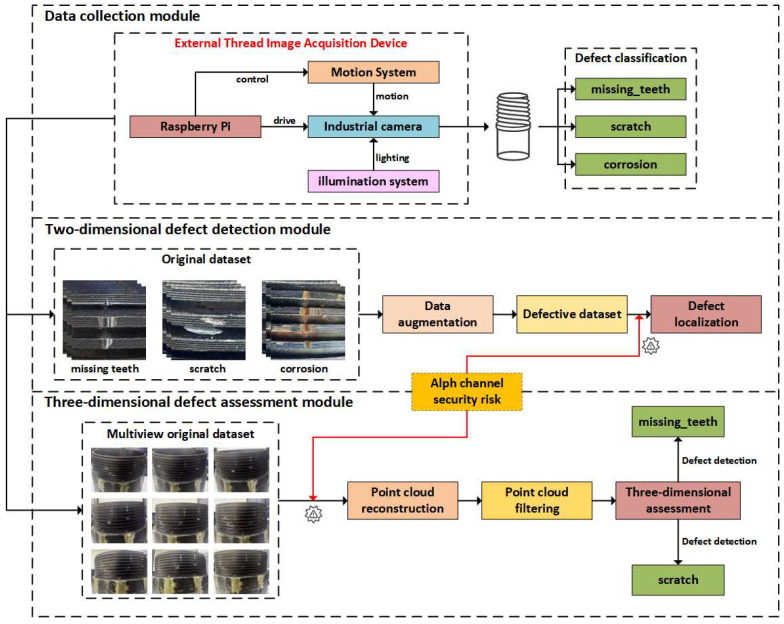
Overview of the Multi-Dimensional External Thread Defect Detection System.

As shown in Figure 1, the main innovations of the external thread defect detection system proposed in this paper are as follows:

(1) A multi-dimensional external thread defect detection framework is proposed, integrating 2D detection and 3D reconstruction to achieve both defect localization and geometric quantification.
(2) A point cloud-based defect analysis method is developed, enabling millimeter-level accuracy in defect size estimation for complex thread structures.
(3) A robustness evaluation is conducted to analyze the impact of image perturbations on detection performance, providing insights for reliable industrial deployment.

## 2. Methodology

### 2.1. Image Acquisition System Hardware Design

**Figure 2:**
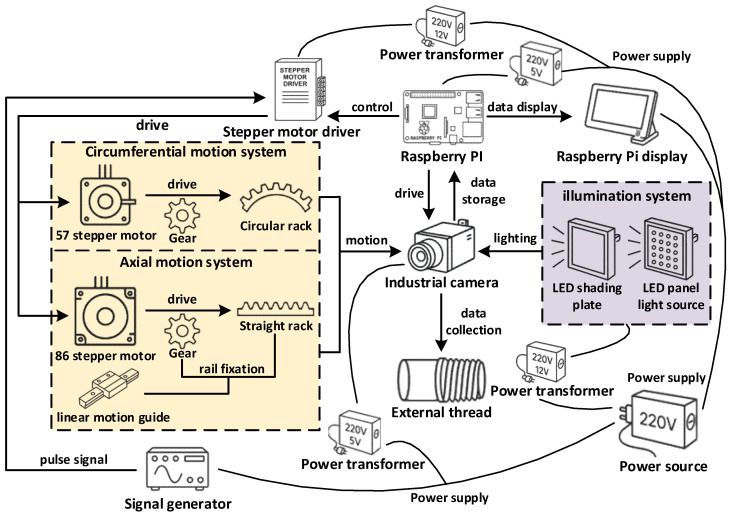
Structure principle diagram of the external thread image acquisition system.

The image acquisition device of this system is carefully designed to achieve high-quality, multi-view image collection of external thread surfaces. The system’s hardware architecture includes mechanical structures, industrial cameras, lighting systems, and control platforms, forming a closed-loop acquisition system. The working principle of the system is shown in Figure 2, covering aspects such as motion control, data acquisition and storage, and light source adjustment.

**Figure 3:**
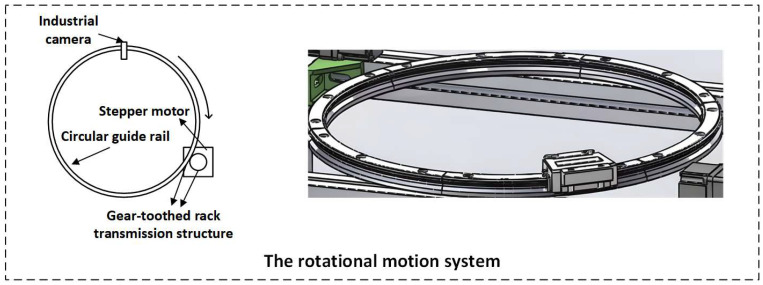
Detailed modeling of the circumferential motion system.

**Figure 4:**
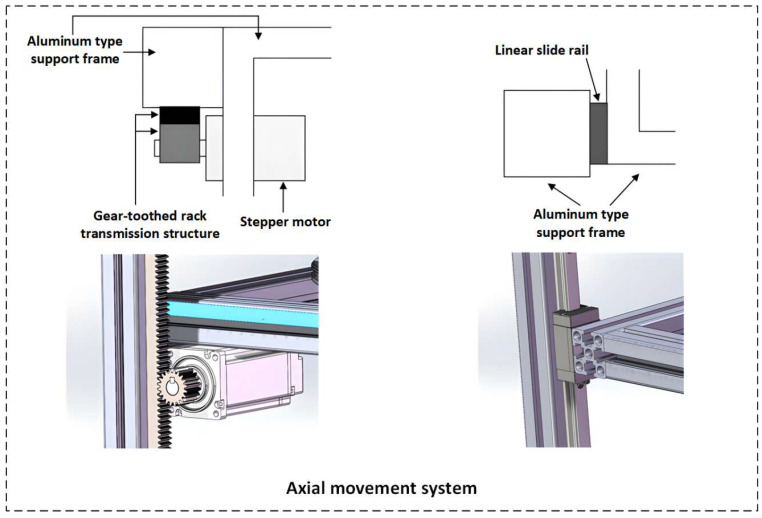
Detailed modeling of the axial motion system.

The mechanical structure module of the system includes a circumferential motion system and an axial motion system (as shown in Figure 3 and Figure 4).

**Figure 5:**
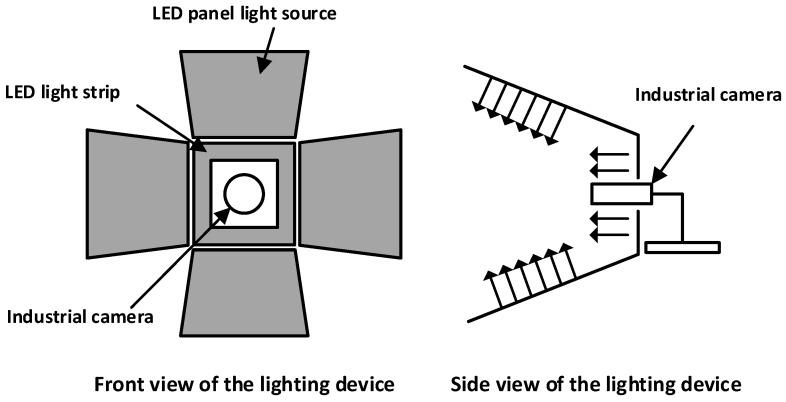
Structure principle diagram of the lighting system.

To ensure uniform lighting and texture restoration during image acquisition, the system employs a composite lighting structure using an LED panel light source and a shading plate (as shown in Figure 5).

**Figure 6:**
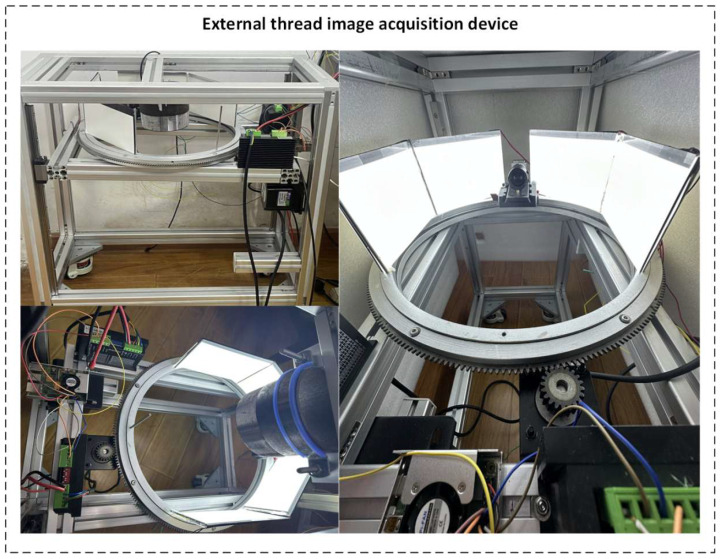
Physical image of the external thread image acquisition system.

As shown in Figure 6, the system is managed by a Raspberry Pi control platform, which is responsible for synchronizing the camera, motion system, and light source control, as well as real-time data storage and transmission. The control functionality of the Raspberry Pi ensures the coordination of all modules during image acquisition, enabling the system to efficiently complete multi-view scanning tasks of the thread surface. The physical diagram of the external thread image acquisition device demonstrates the layout of the entire system’s hardware.

Through the above design, the image acquisition system has been optimized and innovated in several aspects, ensuring the efficiency and stability of the data acquisition process. It provides high-quality raw data for subsequent image data augmentation, 2D defect detection, 3D point cloud reconstruction, and defect recognition.

### 2.2. Image Data Augmentation

The size and diversity of the dataset in external thread defect detection have a decisive impact on the training effect and generalization ability of the YOLO detection model. Due to the scarcity of external thread defect samples and the issue of class imbalance, relying solely on real-world data for training often does not achieve ideal performance. Therefore, image data augmentation techniques play a crucial role in such tasks.

The StyleSAN-XL generation model is a GAN-based (Generative Adversarial Network) image enhancement method, designed to generate high-quality, detail-rich defect images, effectively addressing the issues of sample imbalance and insufficient defect variety. This model combines the advantages of StyleGAN and SAN, allowing the generation of high-resolution images while refining the texture details within the images, providing diverse samples for model training. This, in turn, improves the model’s ability to recognize different defect types and sizes.

Recent studies have shown that adversarial-based data augmentation can improve the robustness of defect detection models by enriching difficult sample distributions [25]. Meanwhile, security-oriented research has revealed that defect detectors may be vulnerable to localized perturbations, such as one-pixel attacks [26], and broader adversarial attack studies further indicate that object detection models can be affected in localization, confidence estimation, and classification simultaneously [27]. Therefore, StyleSAN-XL is designed not only to alleviate sample imbalance and insufficient defect diversity, but also to enhance the detector’s tolerance to complex textures, visual variations, and potential perturbations in industrial inspection scenarios.

Table 1 summarizes the relationships between GAN [28], StyleGAN [29], SAN [30], and StyleSAN-XL and highlights their application and evolution in this study.

To address the instability and mode collapse issues when generating high-resolution images with GAN, StyleGAN introduces significant improvements, especially by incorporating a style control module. The optimization goal of the style control module is:

(1) $$L_{\mathrm{style}\;}=∥F\left(G(z)\right)-F(x){∥}_{2}^{2}$$

where F(x) and $F\left(G(z)\right)$ represent the style features of the real image x and the generated image G(z), respectively. Through minimizing this loss function, the generator can fine-tune and align the style features of the generated image with the real image, ensuring that the visual effect is consistent with the real image.

Although StyleGAN has made significant progress in the quality of generated images, the generation process is still susceptible to instability in the discriminator’s training. To address this, SAN (Self-Attention Network) was introduced as a further optimization of the GAN discriminator. SAN improves the training process of the discriminator by introducing the Slice Optimal Transport (SOT) technique, which enhances the stability and quality of the generated images.

The optimization goal of Slice Optimal Transport (SOT) is:

(2) $$SOT\left(P,Q\right)=\underset{\pi \in \Pi (P,Q)}{inf}\sum_{(x,y)\in \pi }c\left(x,y\right)$$

In this context, $\Pi \left(P,Q\right)$ represents all possible transport plans between distributions P and Q, and $c\left(x,y\right)$ is the transport cost. By optimizing this transport strategy, SAN effectively improves the image’s detail and quality during the generation process.

StyleSAN-XL combines the advantages of StyleGAN and SAN to form a new type of image generation method. The style control module and the optimized discriminator work together, allowing the generator to not only precisely control the style and details of the generated images but also optimize the generation process through a stable discriminator. By combining these elements, StyleSAN-XL can generate high-resolution and detail-rich images, significantly improving the diversity and quality of datasets, especially for data augmentation tasks in small sample scenarios.

### 2.3. Two-Dimensional Defect Detection

**Figure 7:**
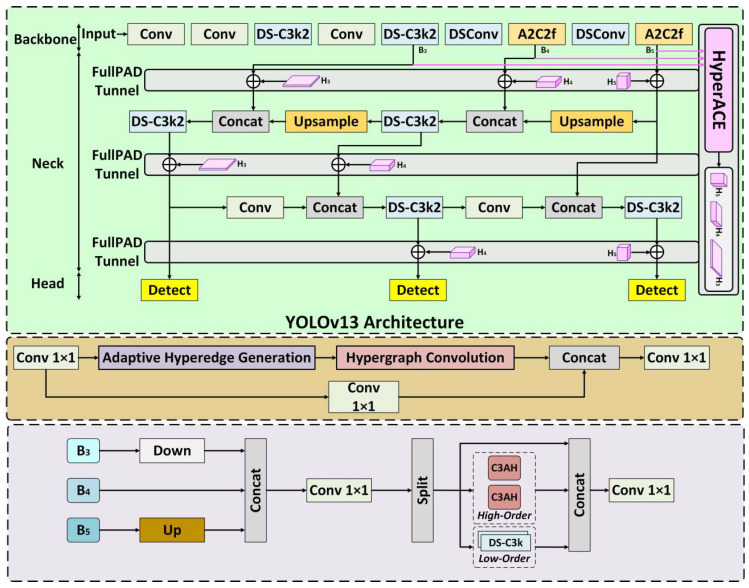
YOLOv13 Network Structure.

In this study’s external thread defect detection system, YOLOv13 [31] was selected as the detector for the 2D detection module, based on its significant advantages in handling complex backgrounds and detecting small defects. The network structure of YOLOv13 integrates three innovative modules: Depthwise Separable Convolution, Hypergraph Convolution, and Adaptive Multi-Scale Feature Fusion. These modules collectively enhance defect detection accuracy and robustness, especially in the detection of the complex geometries of external thread surfaces, showing excellent adaptability. The YOLOv13 network structure is shown in Figure 7.

The adaptability of YOLOv13’s innovative modules to this system is shown in Table 2.

Depthwise Separable Convolution, as one of the key designs of YOLOv13, decomposes the traditional convolution operation into two steps: depthwise convolution and pointwise convolution. This significantly reduces computation and increases inference speed. This design is particularly suitable for small defect recognition tasks in external thread defect detection, enabling fast extraction of key features from images with limited computational resources, especially when detecting small defects such as missing teeth and scratches, ensuring efficient real-time inference and high-precision detection.

YOLOv13 performs exceptionally well in the detection of small objects and in complex background environments, and thus was selected as a 2D defect detector.

### 2.4. 3D Point Cloud Generation and Geometric Reconstruction

In the external thread defect detection task, missing teeth and scratches not only alter the surface texture but also involve geometric fluctuations, containing certain depth information. Relying solely on 2D images for detection and analysis often leads to incomplete recognition and visualization. To overcome this limitation, this study adopts a 3D reconstruction method based on Gaussian Splatting, which provides strong technical support for the precise modeling of external thread geometries.

Gaussian Splatting [32] offers an efficient scene representation approach based on 3D Gaussian distributions and a differentiable rendering mechanism, enabling precise reconstruction of the external thread point cloud. Compared to traditional mesh or voxel-based methods, this approach offers significant advantages in rendering efficiency, geometric continuity, and detail preservation, making it particularly suitable for recovering defect information such as missing teeth and scratches, which have depth features but weak expressions.

**Figure 8:**
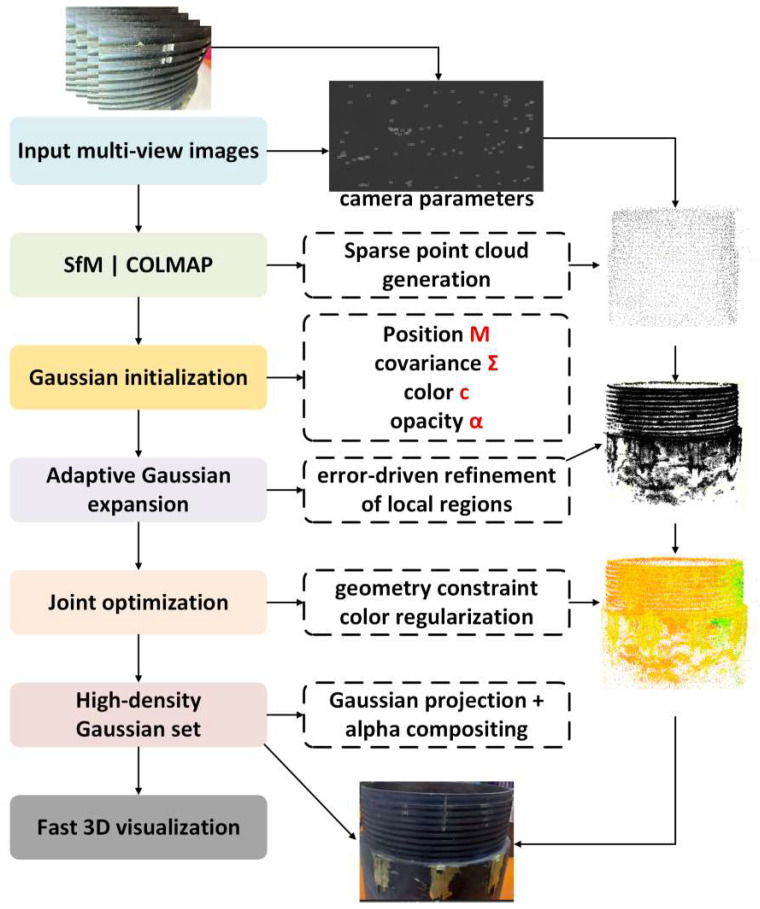
Point Cloud Reconstruction and Rendering Process of External Threads Based on Gaussian Splatting.

As shown in Figure 8, the Gaussian Splatting reconstruction process first generates a sparse point cloud, relying on multi-view geometric constraints. Given 2D projections from different perspectives, triangulation is used to recover 3D points, forming a sparse point cloud that provides global spatial constraints for subsequent modeling. Each sparse point is initialized with a Gaussian distribution, represented as follows:

(3) $$p=\left(c,\sum ,\alpha \right)$$

where p is the spatial position, c is the color, ∑ is the covariance matrix, and α is the transparency. The initial phase of the process ensures the continuity of the geometry within the local region, providing continuity for subsequent optimization of the geometric base.

On this basis, the system performs adaptive high-frequency expansion and refines the reconstruction based on local geometry, which improves the detail representation. If a high-frequency component appears in the first i-th angle projection, the impact error is represented as:

(4) $$e_{i}=∥\begin{array}{c} p_{i}-{\hat{p}}_{i} \end{array}∥$$

where $p_{i}$ is the original point, and ${\hat{p}}_{i}$ is the point calculated by the projection. If the error is small compared to other values, the projection can be partitioned into multiple high-frequency subregions, helping to reduce the projection error and ensuring the smoothness of the reconstructed surface. This approach enhances the efficiency of calculation and construction.

The optimization goal for the final stage of joint optimization, which updates all high-frequency parameters and minimizes the projection error under multiple views, is:

(5) $$L=\sum_{v}{∥\begin{array}{c} I_{v}-{\hat{I}}_{v} \end{array}∥}^{2}$$

where $I_{v}$ is the real image from the v-th view, and ${\hat{I}}_{v}$ is the image synthesized through high-frequency splatting. The process uses the Alpha compositing technique, ensuring correct depth and enhancing the realism of the rendered image.

To prevent color shift and shadow interference, the following optimization is performed to match the color between the images:

(6) $$L_{\mathrm{color}\;}=\sum_{i}\left|c_{i}-{\hat{c}}_{i}\right|$$

where $c_{i}$ is the color value of the i-th high-frequency component, and ${\hat{c}}_{i}$ is the predicted color. This optimization further strengthens the rendering effect, especially in regions with inconsistent lighting.

The rendering efficiency of Gaussian Splatting comes from its method of directly projecting Gaussian ellipsoids onto a 2D plane and performing closed-form accumulation within the local pixel range, which avoids the large-scale volumetric traversal computational overhead present in voxel-based methods. This process is inherently parallel and easy to accelerate with GPUs, greatly improving the efficiency of industrial-grade point cloud reconstruction. Through algorithm optimization, high-quality 3D reconstruction can be achieved using only 2D image data, without the need for high-precision hardware support.

### 2.5. Point Cloud Filtering

During the point cloud reconstruction process, despite the use of a high-precision acquisition system and stable imaging conditions, background interference is still unavoidable. Especially in point cloud reconstruction based on the Gaussian Splatting (GS) algorithm, the system often reconstructs irrelevant background areas (such as support structures and environmental backgrounds) as part of the point cloud data. This redundant information not only affects the quality of the point cloud but also interferes with subsequent defect detection. Therefore, filtering is required to remove redundant background points and local noise from the point cloud.

In point cloud processing, Statistical Outlier Removal (SOR) is a classic filtering method. The basic idea is to determine and remove local outliers by calculating the statistical distance of each point. Specifically, for each point $p_{i}$ in the point cloud, the average Euclidean distance to its k-nearest neighbors is calculated as:

(7) $$d_{i}=\frac{1}{k}\sum_{j=1}^{k}{∥\begin{array}{c} p_{i}-p_{j} \end{array}∥}_{2}$$

where $∥p_{i}-p_{j}{∥}_{2}$ represents the Euclidean distance between point $p_{i}$ and its neighboring point $p_{j}$, and $d_{i}$ denotes the distance of the point $p_{i}$ to its neighboring region. Next, the average distance of all points in the region is calculated, and the global standard deviation of all regions is computed:

(8) $$\mu_{d}=\frac{1}{N}\sum_{i=1}^{N}d_{i},\;\sigma_{d}=\sqrt{\frac{1}{N}\sum_{i=1}^{N}{\left(d_{i}-\mu_{d}\right)}^{2}}$$

where N is the total number of points, $\mu_{d}$ is the mean density of the entire point cloud, and $\sigma_{d}$ is the density fluctuation range.

Finally, outliers are removed according to the following rule for distance determination:

(9) $$\left|d_{i}-\mu_{d}\right|>\alpha \cdot \sigma_{d}$$

where α is the empirical constant. If a point satisfies this condition, it is considered an outlier, and it will be discarded and removed from the point cloud.

However, in the external thread point cloud detection task, directly applying global one-time SOR filtering often results in two issues:

1. Some real points in the main thread geometry may be mistakenly deleted, causing gaps in the thread profile geometry.
2. Floating points near the tooth boundary are not effectively removed, leading to the formation of non-physical peak values during the subsequent slice curvature analysis, which are then misjudged as missing teeth.

**Figure 9:**
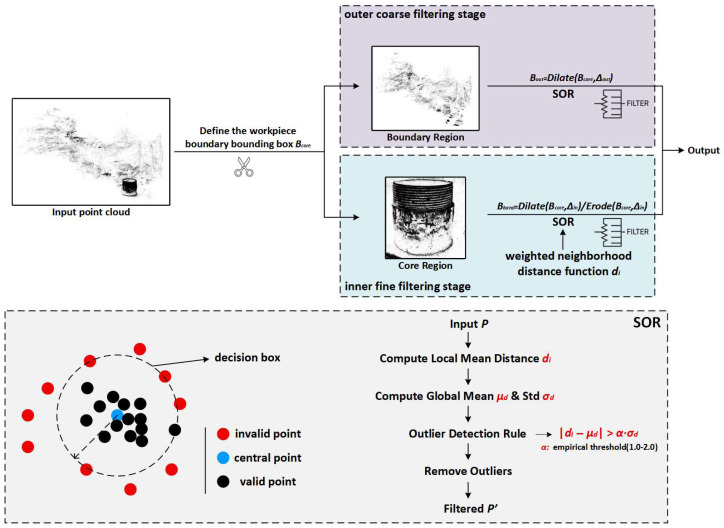
Structural Diagram of the Dual-Constrained Statistical Outlier Removal (DC-SOR) Strategy.

To avoid “false positives” in the external thread body caused by global one-time filtering and to suppress curvature mutations and misdetections caused by boundary redundancy points at the source, this study proposes a Dual-Constrained Statistical Outlier Removal (DC-SOR) strategy (its working principle is shown in Figure 9). After determining the target spatial location of the external thread, the method first performs a coarse filter outside the main 3D region, then applies a fine filter within the main 3D region (including the narrow boundary zone).

In the external layer refinement stage, the workpiece boundary is defined in the 3D space to create a bounding box $B_{core}$, and then the box is expanded into $B_{out}=Dilate\left(B_{core},\Delta_{out}\right)$, leaving only the external points. This expansion preserves the boundary, and the SOR is quickly executed to remove redundant points.

In the internal refinement stage, the boundary of the thread’s top and bottom is constructed with a region boundary band $B_{\mathrm{band}}=Dilate\left(B_{\mathrm{core}},\Delta_{in}\right)\setminus Erode\left(B_{\mathrm{core}},\Delta_{in}\right)$, only operating on boundary points for precise SOR execution. To ensure the boundary point’s preservation ability, we add a region distance metric for internal regions as follows:

(10) $$d_{i}=\frac{1}{k}\sum_{j\in N_{k}(i)}{∥\begin{array}{c} p_{i}-p_{j} \end{array}∥}_{2}\cdot exp\left(-\beta \cdot {∥\begin{array}{c} p_{i}-p_{j} \end{array}∥}_{2}\right)$$

where $\beta \in \left[0,1.0\right]$ is the weight adjustment factor used for fine-tuning the boundary’s contribution to the distance, ensuring the closest points are preserved.

After computing the region distance, the distance metric for each layer and region is determined using the statistical average $\mu ,\mu_{b}$ and standard deviations $\sigma ,\sigma_{b}$, then classified using a threshold:

(11) $$outlier\left(p_{i}\right)=I\left[d_{i}>\mu +\tau \sigma \right],\;{outlier}_{b}\left(p_{i}\right)=I\left[d_{i}>\mu_{b}+\tau_{b}\sigma_{b}\right]$$

where τ and $\tau_{b}$ represent the scaling factors for the external and internal layers, respectively.

In summary, the DC-SOR method builds upon the SOR statistical discrimination mechanism and introduces dual spatial constraints, applying differentiated processing to both the external and internal layers. DC-SOR can maintain the geometric integrity of the external thread while providing cleaner and more stable point cloud input, thereby laying a solid foundation for subsequent 3D defect detection.

### 2.6. Three-Dimensional Defect Detection

#### 2.6.1. Missing Teeth Defect Detection

**Figure 10:**
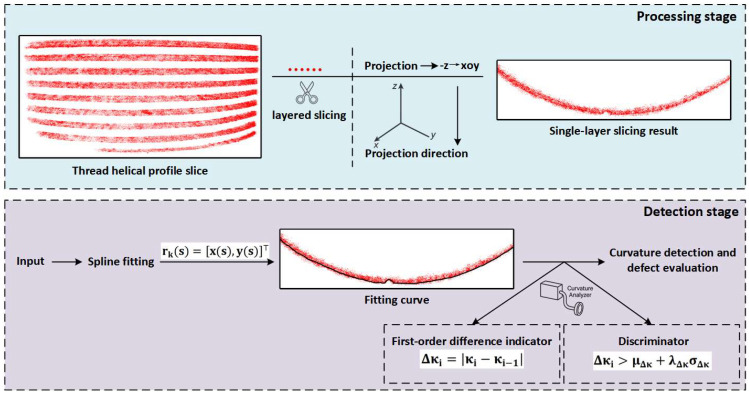
Slice Curvature-Based Tooth-Missing Detection (SCTD) Principle Diagram.

Missing teeth defects on external threads exhibit clear depth information, located along the thread’s helical line, and manifest as axial truncations. In the reconstruction results, when viewed from a direction perpendicular to the xoy-plane, the missing teeth area appears as a significant indentation. This observation is key to detecting the defect using point cloud data. This section proposes a detection method for missing teeth defects based on Slice Curvature Analysis (SCTD), as shown in Figure 10.

Considering that the external thread point cloud data axis is parallel to the z-axis, and the thread’s top and bottom surfaces are parallel to the xoy-plane, horizontal slices are made along the axial direction with a step size of Δz, yielding:

(12) $$P_{k}=\left\{\left(x,y,z\right)\in R^{3}||z-z_{k}|\leq \frac{\Delta z}{2}\right\}$$

The image is projected onto the xoy-plane, resulting in a 2D point set $r_{k}(s)={\left[x(s),y(s)\right]}^{⊤}$. The curvature of this curve is defined as:

(13) $$\kappa (s)=\frac{\left|x^{\prime }(s)y^{\prime \prime }(s)-y^{\prime }(s)x^{\prime \prime }(s)\right|}{{\left(x^{\prime }(s{)}^{2}+y^{\prime }(s{)}^{2}\right)}^{3/2}}$$

where κ(s) represents the curvature at position s along the curve, and it quantifies the curve’s bending intensity. x(s) and y(s) are the coordinates of the curve in the x and y directions, respectively, in the 2D plane. $x^{\prime }(s)$ and $y^{\prime }(s)$ are the first derivatives of the curve’s position, representing the direction of the curve at that point. $x^{\prime \prime }(s)$ and $y^{\prime \prime }(s)$ represent the second derivatives of the curve, indicating the rate of change of the curve’s bending.

To capture the variation in curvature, the first-order difference is defined as:

(14) $$\Delta \kappa_{i}=\left|\kappa_{i}-\kappa_{i-1}\right|$$

where $\Delta \kappa_{i}$ represents the magnitude of curvature change, reflecting the extent to which the curve undergoes bending or abrupt changes at the segment boundary. $\kappa_{i}$ is the curvature value at the current segment, indicating the bending intensity of the curve at that segment, while $\kappa_{i-1}$ is the curvature of the previous segment, showing the bending intensity of the curve at the previous position. This difference calculation can effectively capture the abrupt or transitional characteristics in the curvature variation, identifying sudden changes or bends in the curve.

When the value of Δκ in the continuous segments exceeds the statistical threshold, it indicates that the tooth profile at that position exhibits a break or defect. Specifically, let $\mu_{\Delta \kappa }$ and $\sigma_{\Delta \kappa }$ represent the global mean and standard deviation of Δκ, and the following condition is established:

(15) $$\Delta \kappa_{i}>\mu_{\Delta \kappa }+\lambda_{\Delta \kappa }\sigma_{\Delta \kappa }$$

When this condition is met, it is determined as a candidate region for missing teeth defects. Through inter-layer aggregation and spatial position remapping, the missing teeth location can be accurately identified in 3D space.

#### 2.6.2. Scratch Defect Detection

Scratch defects typically occur in the groove region between the top and bottom of the thread, with depth information present but not significant. They are visually represented as transverse cutting states. In the Gaussian Splatting point cloud reconstruction results, these defects correspond to an abnormally dense point cloud distribution in the groove region, which is key to detecting the defect using point cloud data.

**Figure 11:**
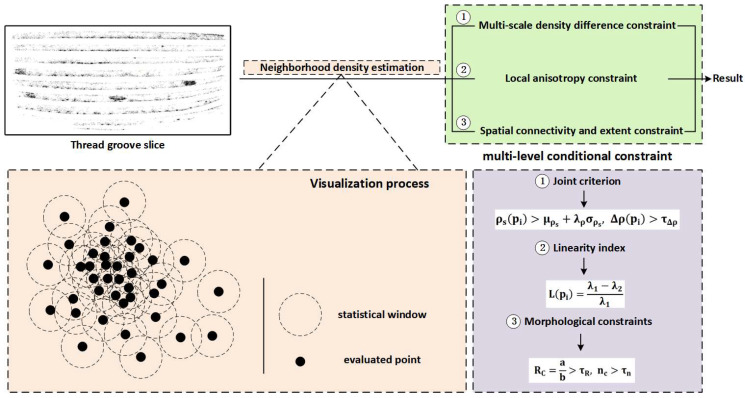
Local Point Density Estimation (LPDE) Detection Principle Diagram.

This section designs a detection method based on Local Point Density Estimation (LPDE), and its working principle is shown in Figure 11, aiming to detect external thread scratch defects based on point cloud data.

Let the slice point set of the groove region be:

(16) $$P=\{p_{i}{\}}_{i=1}^{N}$$

In the region, the point density is calculated by defining a neighborhood with a radius r as $N_{r}\left(p_{i}\right)$:

(17) $$\rho \left(p_{i}\right)=\frac{\left|N_{r}\left(p_{i}\right)\right|}{\pi r^{2}}$$

where $\left|N_{r}\left(p_{i}\right)\right|$ represents the number of points within a region centered at $p_{i}$ with radius r, and $\pi r^{2}$ is the area of the region (in the slice plane). The function (17) represents the local point density at each point. For all points in the dataset, the density is calculated to obtain the average value $\mu_{\rho }$ and standard deviation $\sigma_{\rho }$.

When a point satisfies:

(18) $$\rho \left(p_{i}\right)>\mu_{\rho }+\lambda_{\rho }\sigma_{\rho }$$

It is classified as an outlier in a high-density region, which is marked as a location for potential defect detection. Here, $\lambda_{\rho }$ is a scaling factor.

However, in practical detection, we found that using only local point density as the criterion for scratch detection leads to insufficient robustness. Specifically, when small regions with abnormally high point density appear in non-scratch areas, they are easily misclassified as scratch defects because their local statistical density $p_{i}$ is much higher than the global mean. These point sets typically contain only a few hundred points and cover a very small spatial area. Although they exhibit high-density characteristics, they lack the spatial continuity, extension, and band-like distribution that scratches exhibit. In contrast, real scratch defects often present as point clusters with medium to high density and more extensive spatial coverage. Therefore, relying solely on density thresholds for detection makes it difficult to distinguish between “local high-density noise” and “continuous scratch structures”.

To address this issue, this paper introduces three additional constraints based on the original density criterion: multi-scale density differences, spatial continuity, and morphological anisotropy, in order to achieve more robust scratch detection.

First, multi-scale analysis is used to suppress the impact of high-density noise in very small regions.

Let the radius of the small-scale neighborhood be $r_{s}$ and the radius of the large-scale neighborhood be $r_{l}$. The average densities for these two scales are calculated as follows:

(19) $$\rho_{s}\left(p_{i}\right)=\frac{\left|N_{r_{s}}\left(p_{i}\right)\right|}{\pi r_{s}^{2}},\;\rho_{l}\left(p_{i}\right)=\frac{\left|N_{r_{l}}\left(p_{i}\right)\right|}{\pi r_{l}^{2}}$$

The density difference is defined as:

(20) $$\Delta \rho \left(p_{i}\right)=\rho_{s}\left(p_{i}\right)-\rho_{l}\left(p_{i}\right)$$

When the point belongs to an extreme local noise cluster, $\rho_{s}$ is high, but $\rho_{l}$ increases rapidly, and $\Delta \rho \left(p_{i}\right)$ approaches zero. In contrast, areas with scratch-like features in larger regions maintain a higher density in $\rho_{s}$, while $\rho_{l}$ differs significantly.

Therefore, this paper adopts a combined judgment criterion:

(21) $$\rho_{s}\left(p_{i}\right)>\mu_{\rho_{s}}+\lambda_{\rho }\sigma_{\rho_{s}},\;\Delta \rho \left(p_{i}\right)>\tau_{\Delta \rho }$$

where $\tau_{\Delta \rho }$ is an empirical constant used for filtering local noise points.

Scratches exhibit obvious linear and directional consistency in local structures. This system incorporates anisotropic constraints.

For each selected point neighborhood $N_{r_{s}}\left(p_{i}\right)$, a Principal Component Analysis (PCA) is performed. Let the eigenvalues satisfy $\lambda_{1}\geq \lambda_{2}\geq \lambda_{3}$, and the linearity measure is defined as follows:

(22) $$L\left(p_{i}\right)=\frac{\lambda_{1}-\lambda_{2}}{\lambda_{1}}$$

When $L\left(p_{i}\right)$ is large, the point cloud primarily aligns along one direction and exhibits a slender structure; conversely, when $L\left(p_{i}\right)$ is small, the point cloud shows a more isotropic distribution, indicating that the points belong to a flat or background region.

To further ensure spatial continuity and consistency, spatial connectivity and boundary constraints are added. Specifically, a density-based clustering algorithm (DBSCAN) is applied to the point set defined by functions (19) and (20), and the main axes of each connected component are calculated based on the length a, the short axis b, and the number of points included $n_{c}$.

The defined geometric constraint is as follows:

(23) $$R_{C}=\frac{a}{b}>\tau_{R},\;n_{c}>\tau_{n}$$

Here, $R_{C}$ represents the aspect ratio, $\tau_{R}$ is the morphological threshold, and $\tau_{n}$ is the minimum number of connected points. This constraint effectively excludes high-density clusters with very few points or small areas, retaining only stripe-shaped scratch structures with sufficient length and point count.

Through the above three-layer constraints, this paper establishes a robust scratch recognition mechanism based on ‘density difference–directional consistency–spatial continuity’. This method not only identifies scratches with a distinct linear morphology but also effectively suppresses misjudgments caused by high-density noise points in very small areas, significantly improving the stability and accuracy of external thread scratch detection.

## 3. Experiment

### 3.1. Data Augmentation

**Figure 12:**
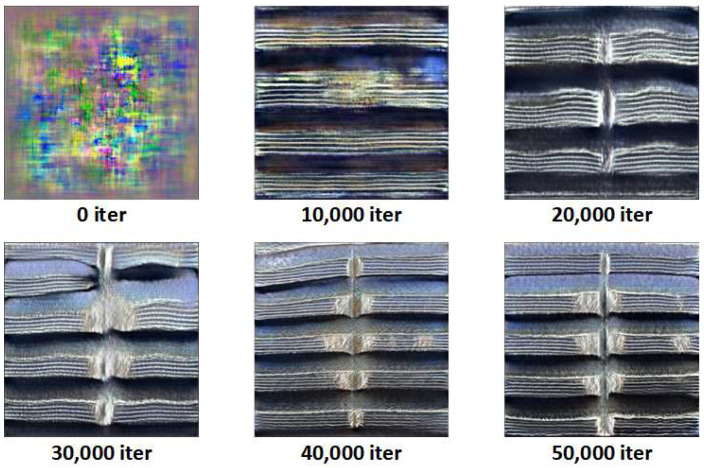
Generation of Missing Teeth Defects.

**Figure 13:**
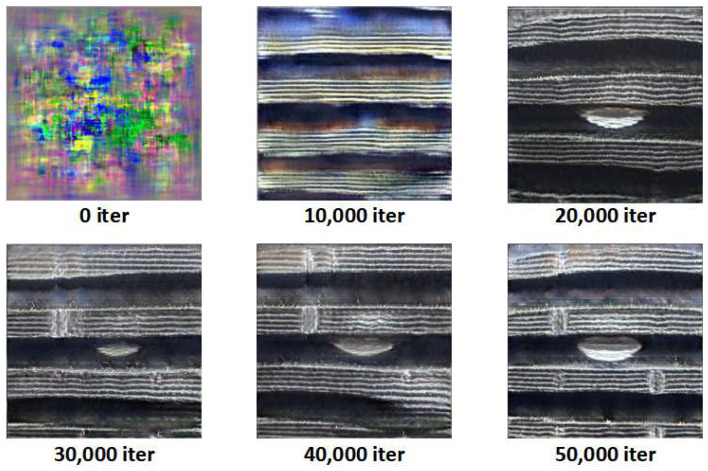
Generation of Scratch Defects.

**Figure 14:**
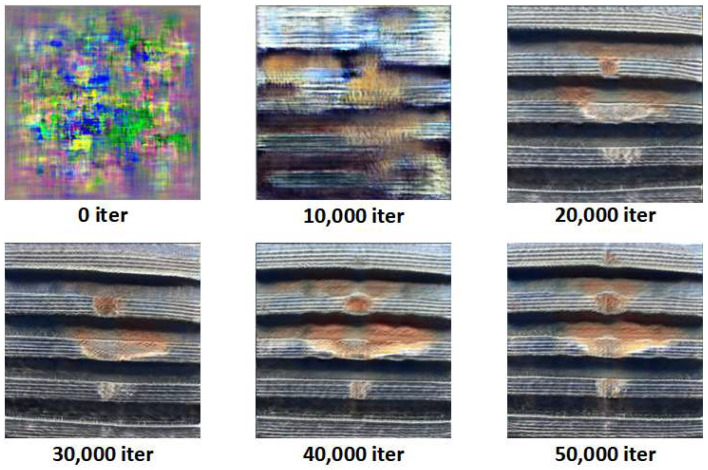
Evolution of Corrosion Defects.

This experiment uses StyleSAN-XL to synthesize images of three typical external thread defects: missing teeth, scratches, and corrosion. As the training iterations progress, the generated samples gradually evolve from unstructured noise into thread images with clearly defined defect morphologies. Figure 12, Figure 13 and Figure 14 show representative samples from different stages of iteration.

Figure 12 shows the generation results for missing teeth defects: starting from the colored noise at 0 iterations, to the initial appearance of thread stripes at 10,000 iterations, and finally, at 30,000–50,000 iterations, the missing teeth structure takes form with clear contours and breakage, with the surface texture of the teeth highly resembling real samples.

Figure 13 presents the generation process of scratch defects: in the early samples, scratches appear only as blurred streaks. As the iterations increase, the length, directionality, and depth of the scratch stabilize. By the 50,000th iteration, the scratch exhibits linear damage patterns similar to real scratches.

Figure 14 shows the evolution of corrosion defects: initially, irregular color blocks appear, which gradually evolve into rust spots with blurred boundaries. At 30,000–50,000 iterations, the corrosion areas develop into regions with irregular spatial distribution and natural light-dark transitions, with the overall texture closely resembling real corrosion.

The synthesized defect images are incorporated into the training set of the YOLOv13 detector as part of the training samples, compensating for the limitations of real-captured data in terms of class distribution and morphological variability, and thereby enhancing the diversity of external thread defect samples at the data level.

**Figure 15:**
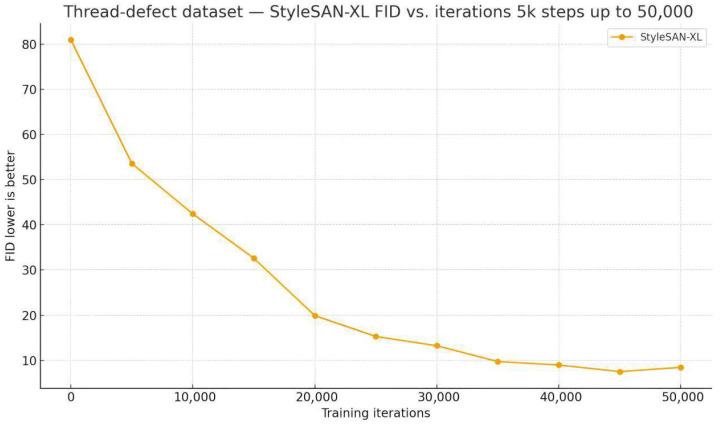
FID Curve of StyleSAN-XL Training for External Thread Defect Generation.

To quantitatively evaluate how well the generated images approximate the real data distribution, this experiment adopts the Fréchet Inception Distance (FID) as the quality metric (lower values indicate that the generated distribution is closer to the real one). Figure 15 plots the FID as a function of training iterations: it decreases monotonically from about 80 in the initial stage to around 10 at 50,000 iterations, indicating that StyleSAN-XL progressively converges towards the statistical distribution of real external thread defect images during training.

For dataset construction, the YOLOv13 training set consists of both real and synthetic samples. Real images are captured by the dedicated external-thread imaging device and contain three defect categories—missing teeth, scratches, and corrosion—with 200 images per category, for a total of 600 images. On this basis, StyleSAN-XL is trained separately for each defect type up to 50,000 iterations, and 5000 synthetic images are generated per category, yielding 15,000 synthetic samples. The final training dataset thus comprises 15,600 images in total, including 600 real and 15,000 synthetic images.

### 3.2. Two-Dimensional Defect Detection

After completing the data augmentation and dataset construction for external thread defects, this section presents 2D defect detection experiments using YOLOv13 for three types of defects: missing teeth, scratches, and corrosion. The model’s performance is evaluated from three aspects: detection visualization results, evaluation metrics curves, and training convergence.

**Figure 16:**
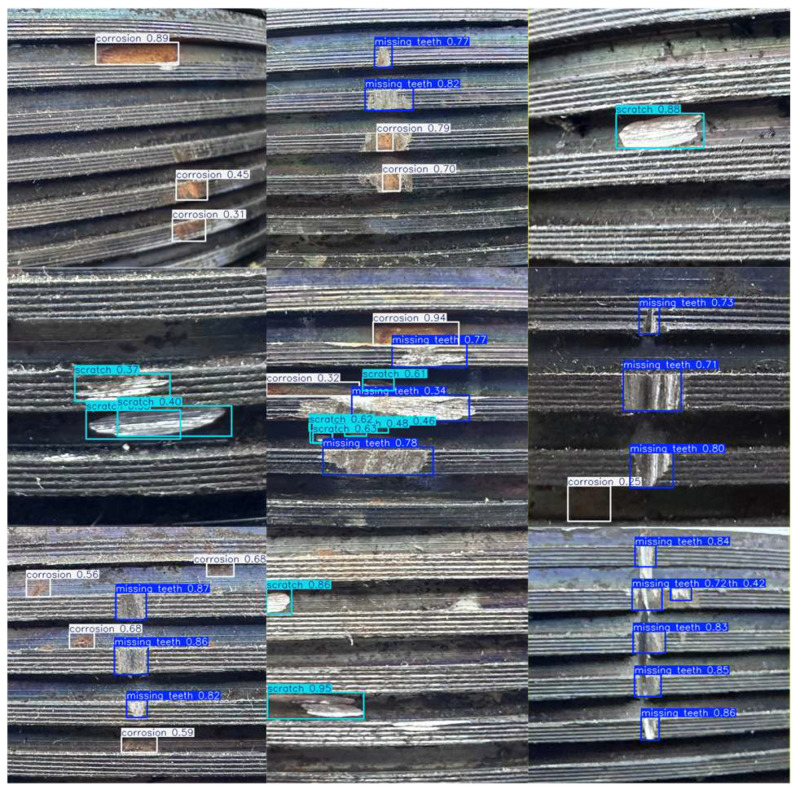
Detection Results for External Thread Defects on Test Set.

Figure 16 presents some detection results on the test set. It can be observed that all three defect types are detected stably: the missing teeth defect forms compact rectangular detection boxes at the thread crest or valley; the scratch defect appears as elongated targets distributed along the thread axis or spiral direction; the corrosion defect is concentrated in local regions with rough surface texture and dark or yellowish color. Each detection box is accompanied by its respective confidence value, with the confidence for missing teeth and scratches generally higher than 0.8. The confidence for corrosion, in complex backgrounds, is slightly lower, but the overall target boundary and classification remain reasonable, indicating the model’s good localization and classification ability in multi-defect coexistence scenarios.

**Figure 17:**
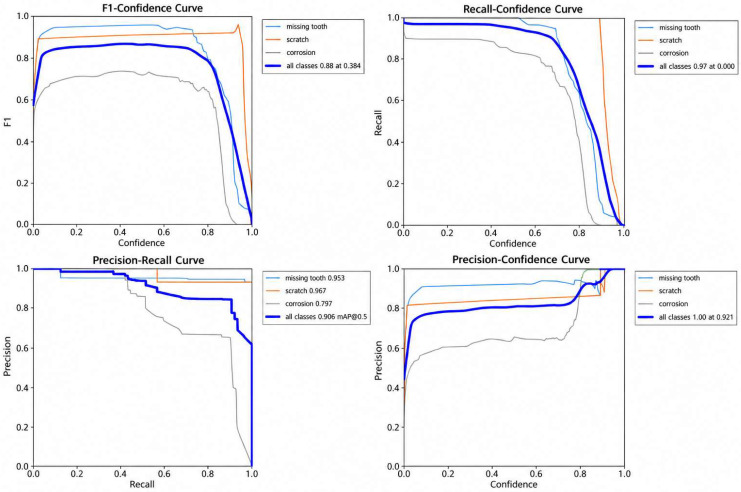
Evaluation Curves for F1-Confidence, Recall-Confidence, Precision-Recall, and Precision-Confidence on Test Set.

Figure 17 shows the F1-Confidence, Recall-Confidence, Precision-Recall, and Precision-Confidence curves calculated based on the test set.

The quantitative evaluation results demonstrate that the proposed model achieves stable detection performance across different external thread defect categories. As shown in Table 3, the overall F1-score reaches approximately 0.88, while the recall achieves nearly 0.97 at a moderate confidence threshold (~0.38), indicating that the model can effectively balance precision and recall through appropriate threshold selection. From the precision–recall analysis, the AP@0.5 values for Missing Tooth and Scratch defects reach 0.953 and 0.967, respectively, demonstrating strong feature extraction capability for structural and surface damage defects. In comparison, the Corrosion category achieves a relatively lower AP@0.5 of approximately 0.797, mainly due to its irregular morphology, weak texture boundaries, and higher intra-class variability under complex illumination conditions.

Nevertheless, the proposed method still maintains a satisfactory overall mAP@0.5 of approximately 0.906, confirming its robustness and effectiveness in multi-type external thread defect detection tasks.

**Figure 18:**
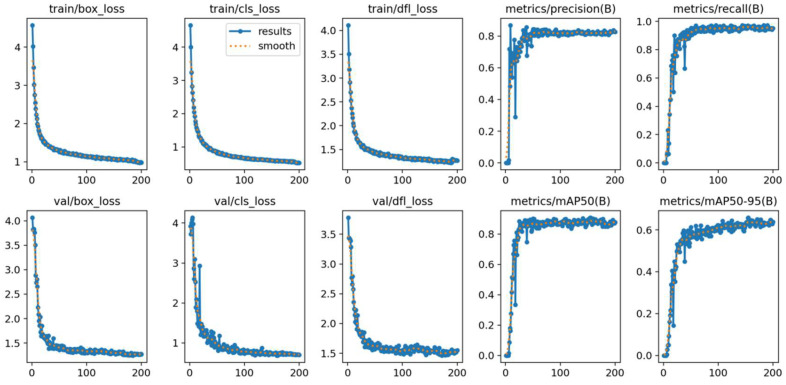
Training and Validation Losses and Performance Metrics (Precision, Recall, mAP) vs. Epochs.

Figure 18 displays the curves of losses and performance metrics during the training and validation phases, including train/val box_loss, cls_loss, dfl_loss, precision, recall, mAP@0.5, and mAP@0.5–0.95.

To further demonstrate the superiority of YOLOv13, as shown in Table 4, an ablation analysis was conducted on the GFLOPs, mAP, parameter quantity, and model size of all previous YOLO versions. Although YOLOv5n has a lower model complexity, its detection accuracy is relatively limited; YOLOv6n improves the detection performance by enhancing the network width and feature extraction capabilities, but its GFLOPs, parameter quantity, and model size increase significantly, resulting in an increase in inference delay. In contrast, YOLOv8n and YOLOv9t maintain a lower computational cost while further improving the detection accuracy. Among them, YOLOv9t achieves a high mAP with only 2.1 M of parameters, demonstrating superior parameter utilization efficiency. Further, YOLOv11n, YOLOv12n, and YOLOv13n exhibit better accuracy-efficiency balance in network structure optimization and lightweight feature fusion. Their GFLOPs are all controlled within the range of 6–7 G, while maintaining high detection accuracy and low inference delay. However, YOLOv13n achieves the optimal comprehensive balance among model parameters, computational complexity, and inference speed, indicating that the new generation of lightweight detectors has stronger application potential in edge deployment and real-time remote sensing target detection tasks.

In conclusion, based on visualization results, evaluation metrics, and training analysis, YOLOv13 achieves satisfactory detection performance in the 2D detection task for missing teeth, scratches, and corrosion defects on external threads. It is capable of outputting high-confidence defect candidate regions under complex textured backgrounds and multi-defect coexistence conditions. This 2D detection module provides reliable defect ROI input for subsequent 3D reconstruction and geometric feature analysis based on point clouds, making it a key front-end component in the entire external thread defect detection system.

Table 5 reports YOLOv13 detection performance with increasing numbers of synthetic images (real images fixed at 600). Performance consistently improves as the synthetic-to-real ratio increases. The baseline model (S0) achieves Precision 0.842, Recall 0.801, and mAP@0.5 0.817. With the full augmented dataset (S4, 15,000 synthetic images), the model reaches Precision 0.89, Recall 0.97, and mAP@0.5 0.906, matching the final YOLOv13n results. Performance gains gradually saturate beyond a 10:1 ratio, indicating that excessive synthetic data yields diminishing returns without degrading results. This demonstrates that StyleSAN-XL-generated images effectively enrich the dataset and justify the use of GAN-based augmentation for small-sample industrial defect detection.

### 3.3. Point Cloud Reconstruction

After completing the 2D defect detection task, this section validates the 3D detection method. The point cloud reconstruction of external thread workpieces is performed using the GS reconstruction algorithm, and the inherent advantages of GS in adapting to the external thread surface reconstruction are analyzed.

**Figure 19:**
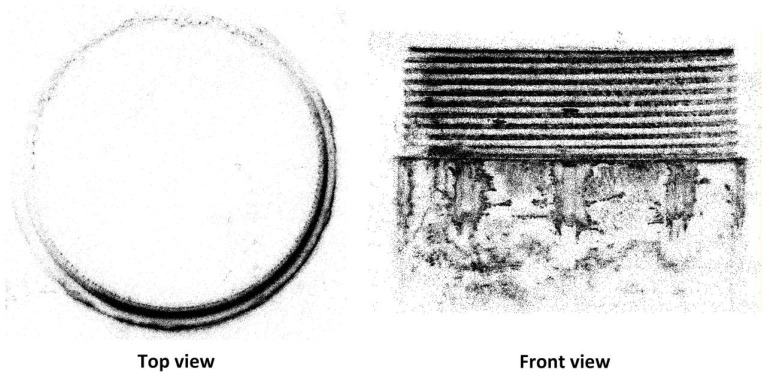
Top and Front Views of GS-Reconstructed Point Cloud for External Thread Workpiece.

Figure 19 presents the top view and front view of the external thread workpiece based on the GS-reconstructed point cloud. In the top view, the outer diameter of the external thread is represented as a continuous, closed high-density annular point band, with clear internal and external boundary definitions and good roundness, indicating that the cylindrical outer profile is effectively preserved during reconstruction. In the front view, the thread profile forms multiple approximately parallel strip-shaped point clouds along the axial direction, with clear layering between the crest and root, and higher point cloud density in the surface’s undulating regions. The longitudinal geometric details are clearly discernible, indicating high geometric fidelity in the overall thread profile and local depth undulations during reconstruction.

It is important to note that the point cloud density along the circumferential direction in the top view is not uniform: high-density point bands are formed only in certain circumferential sections, while other areas are relatively sparse. This density variation is not a limitation of the reconstruction algorithm, but rather a result of the input data’s coverage. This study did not perform a 360° full circumference reconstruction; instead, based on the earlier 2D defect detection results, only a small range of thread images containing defects and their neighboring regions were selected as input for point cloud reconstruction. For these regions, where the 2D detection marked the defects, the view angles were sufficient, with high overlap and complete depth constraints, allowing the point cloud to be restored with high density and detail. In contrast, areas not selected as reconstruction targets lacked adequate viewing angle constraints, and their corresponding point clouds naturally appeared as sparse distributions.

**Figure 20:**
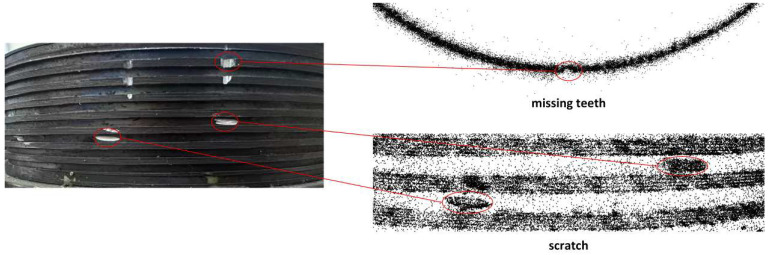
Local Point Cloud Features for Missing Teeth and Scratch Defects from GS Reconstruction.

This local reconstruction strategy, based on 2D priors, not only explains the density distribution characteristics of the thread point cloud in the Figure 20 but also constitutes a key advantage of this method: by performing high-density 3D reconstruction only in the target defect areas, it significantly reduces the reconstruction area, thereby lowering memory consumption and computation time. On the other hand, it achieves higher point cloud resolution and clearer depth details in the focus areas, providing high-quality geometric data support for subsequent millimeter-scale external thread defect 3D detection.

The optimization goal of GS is to minimize rendering error through an adaptive densification mechanism that dynamically adjusts the Gaussian distribution’s density, responding to geometric changes and photometric differences based on position gradients. To further demonstrate GS’s restoration of defect details, this section slices the reconstructed point cloud based on the 2D defect detection results for missing teeth and scratches, as shown in Figure 20.

For the missing teeth defect, which has significant depth variation and complex local geometry, GS effectively captures these subtle depth changes. Despite the millimeter-scale depth of the missing teeth defect, its noticeable depth difference compared to the surrounding flat areas allows GS to accurately restore the geometry and depth information of the gap. Although reconstructing millimeter-scale depth gaps is inherently challenging, GS, with its algorithmic advantages, successfully reconstructs these depth details based on 2D image input.

For the scratch defect, which has shallower geometric depth, GS still effectively captures and reconstructs the scratch area. The scratch area shows significantly highlighted metallic gloss, while the surrounding flat regions appear dark in tone. This strong photometric contrast enhances the point cloud density at the scratch area, allowing for accurate reconstruction of the shallow depth information. By sensitively responding to photometric differences, GS is able to efficiently restore defect details even with minor geometric undulations, confirming its exceptional capability in image-based defect reconstruction.

**Figure 21:**
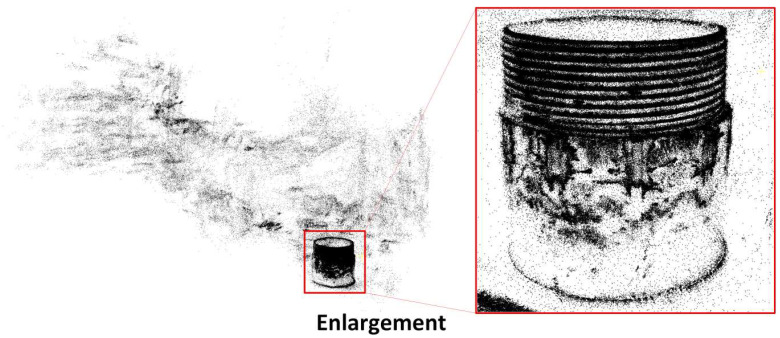
Point Cloud Reconstruction Using Gaussian Splatting (GS) Algorithm, Highlighting Noise in Non-Defect Regions.

Based on the above content, and as shown in Figure 21, it is important to note that the original point cloud reconstruction using the Gaussian Splatting (GS) algorithm contains a significant amount of noise. This noise originates primarily from areas outside the scope of the focused defect regions, where the system’s point density is not as controlled. As shown in the reconstructed point cloud, scattered points in the background and areas outside the target defects lead to irregularities in the overall quality of the point cloud. Although GS excels in reconstructing detailed geometries in defect regions, such as missing teeth or scratches, the surrounding regions exhibit lower point density, resulting in incomplete or noisy areas within the reconstruction. This emphasizes the importance of limiting the reconstruction scope to the defect zones identified in the 2D detection phase, as it improves the quality of the point cloud by concentrating resources on the most relevant regions.

### 3.4. Point Cloud Filtering

In this experiment, a hierarchical filtering strategy based on DC-SOR is used to optimize the external thread point cloud. The optimization objective is to suppress redundant background and noise point clouds while preserving the thread’s geometric details, thus improving the accuracy of subsequent 3D detection and defect recognition.

**Figure 22:**
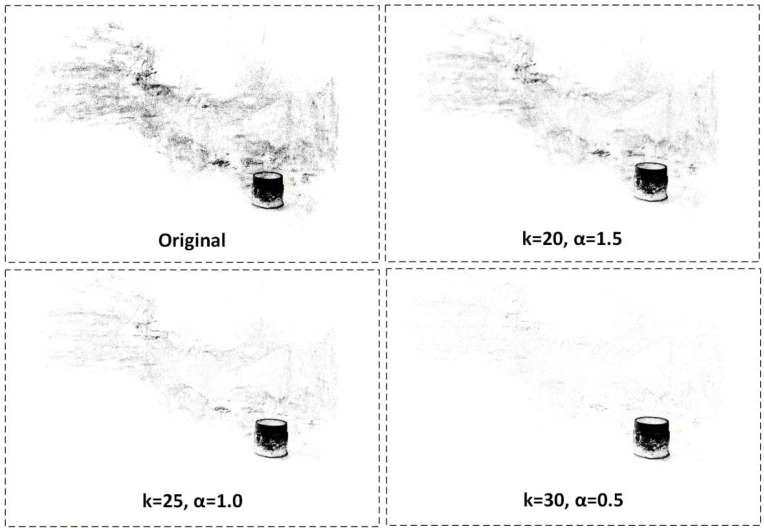
Outer-Layer Filtering Results of External Thread Point Cloud.

Figure 22 shows the visualization results of the outer-layer filtering. Since high-precision reconstruction is performed using Gaussian Splatting, the original point cloud contains not only the surface information of the external thread but also redundant points from unrelated areas, such as the workpiece support structure and the environmental background. These redundant point clouds can significantly interfere with the 3D analysis of the thread target. To address this, the outer-layer filtering first applies spatial constraints to the thread region and then progressively suppresses background point clouds that are further away from the main body through a three-stage filtering gradient (from weak to strong). As the filtering strength increases, the background point clouds are gradually removed, while the geometric contour of the thread body remains continuous and intact, achieving the expected “background removal–body preservation” effect.

**Figure 23:**
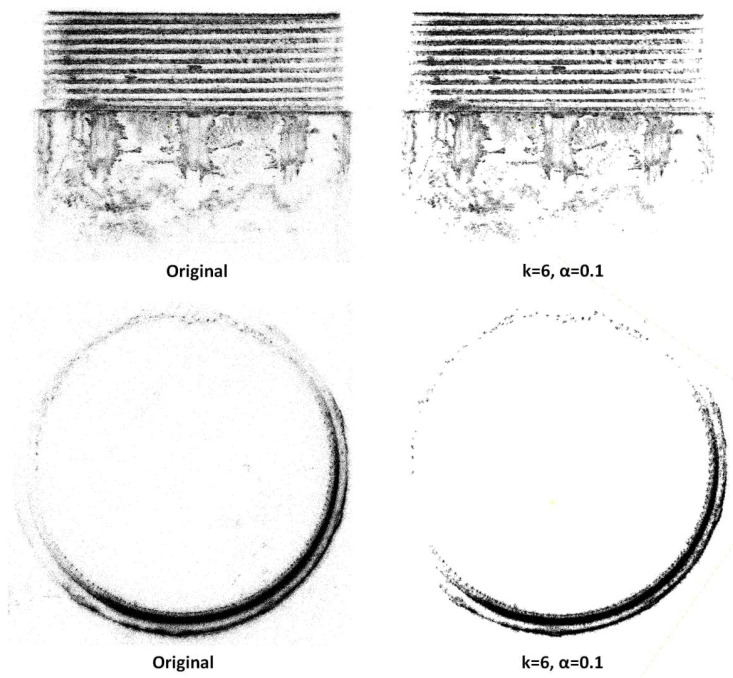
Inner-Layer Filtering Results of External Thread Point Cloud.

Figure 23 shows the effects of the inner-layer filtering. After completing the outer-layer filtering, some local noise, such as burr points and floating points, may still remain. These noises not only affect the visual clarity of the thread structure but may also interfere with subsequent curvature analysis and defect detection. To further improve point cloud quality, the inner-layer filtering applies refined DC-SOR constraints to key areas such as the crest and trough of the thread, selectively removing local outliers while maximizing the preservation of the thread boundary. After this phase of processing, the point cloud distribution on the thread surface becomes more compact, and the boundary transitions become smoother, providing purer and more stable input for subsequent 3D feature extraction.

**Figure 24:**
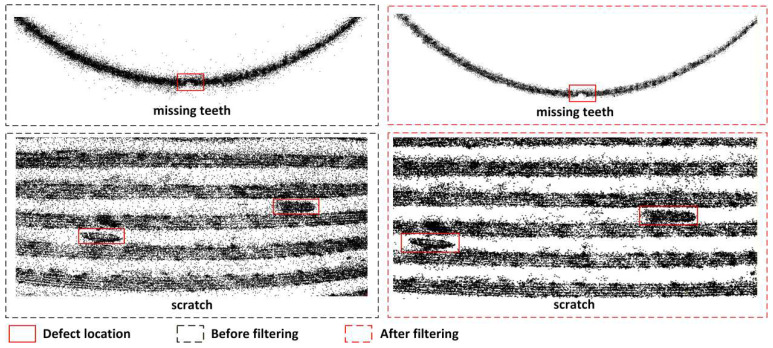
Comparison of Point Cloud Slices Before and After Filtering for Defect Regions.

Figure 24 provides a comparison of the point cloud slices before and after filtering for typical defect regions. Before filtering, due to the overlap of noise and redundant point clouds, the contours of the missing teeth and scratch areas appear blurred, making it difficult to accurately identify the defect boundaries. After filtering, the geometric contours of the tooth gap and the scratch in the trough are significantly enhanced, with clear and continuous boundaries, and the contrast between the defect areas and the normal tooth shape is greatly improved.

Through the combination of coarse outer filtering and fine inner filtering using the DC-SOR strategy, we have achieved phased purification of the external thread point cloud, from global background to local boundaries. This method not only ensures the integrity of the thread’s geometric structure but also significantly enhances the discernibility of defect areas.

### 3.5. Three-Dimensional Defect Detection

**Figure 25:**
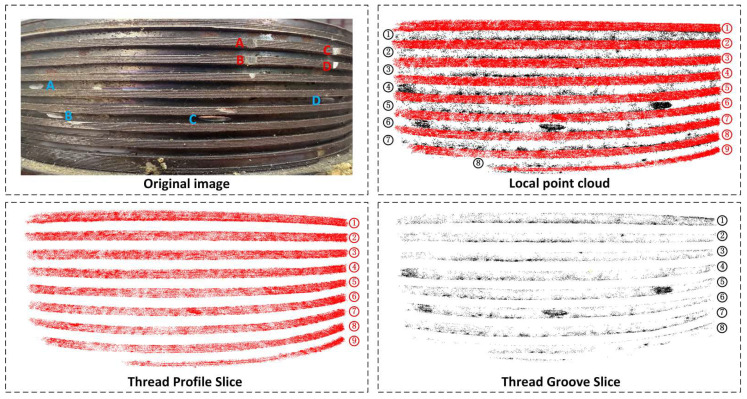
Thread Profile and Groove Region Slices.

After completing the 2D defect detection tasks, this section focuses on validating the 3D detection method. First, the local external thread point cloud is axially sliced and divided into thread profile regions (Thread Profile Slice) and groove regions (Thread Groove Slice), as shown in Figure 25. The thread profile slices cover the thread crest and valley contours and are used as input for detecting missing teeth. The groove slices correspond to the groove area between the upper and lower threads, used as input for scratch detection. This spatial partitioning confines different defect types within more sensitive geometric regions, reducing mutual interference and providing a stable data platform for subsequent detection algorithms.

**Figure 26:**
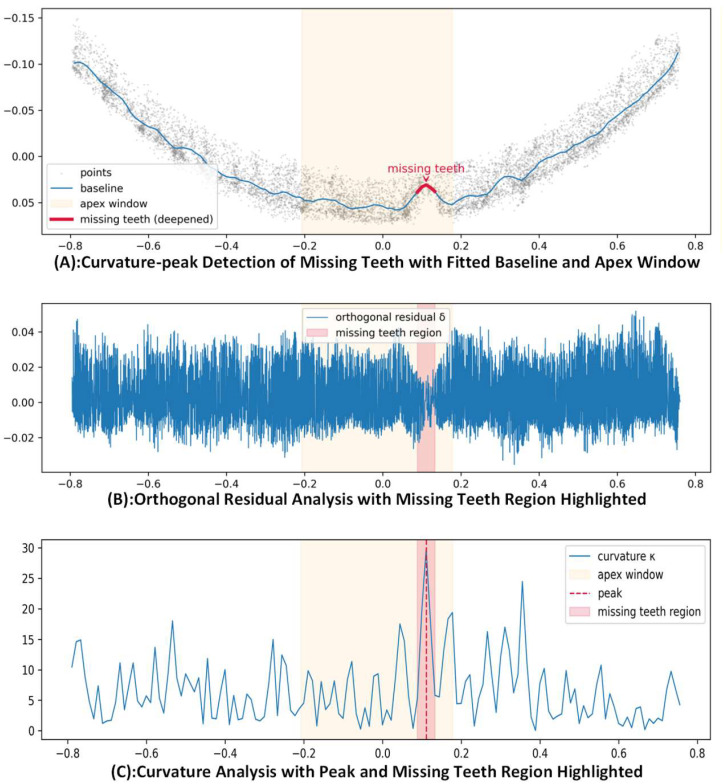
Detection and Analysis of Missing Teeth Defect in Thread Profile Slice 2: Curvature, Orthogonal Residuals, and Curvature Peak.

Taking Thread Profile Slice 2 as an example, SCTD detection is applied to the missing teeth region (C) on its right side. Figure 26 shows the complete detection results.

Figure 26A illustrates the fitted baseline and point cloud distribution of Slice 2 in the curve coordinate system. The thread profile is continuous and smooth in a “U” shape, with a noticeable depression on the right side. The baseline near the missing teeth is highlighted and located within the apex window, indicating that the depression satisfies the curvature mutation condition and falls within the reasonable spatial range of the valley. The scattered point cloud contracts inward at the missing teeth, forming a geometrical indentation consistent with the visual gap, achieving consistent defect representation from the 3D point cloud to the 2D slice curve.

Figure 26B shows the distribution of orthogonal residuals as the curve coordinate changes. Except near the missing teeth, the residuals oscillate slightly around zero, indicating that the fitted baseline accurately describes the average profile of the normal thread. However, in the missing teeth region, the residuals show a continuous offset, and the extreme value width matches the deep segment height of the missing teeth in Figure 26A. This local anomaly in the residual distribution further confirms that the region has a stable geometric loss in the normal direction, not isolated noise or fitting error.

Figure 26C presents the curvature κ distribution of the same curve. A sharp peak is seen at the missing teeth location, with a magnitude significantly higher than the background fluctuations at other positions. This peak is enclosed by the apex window and the missing teeth region, thereby avoiding isolated noise responses far from the valley. Combining the geometric and residual information from Figure 26A,B confirms that the missing teeth on the right side of Thread Profile Slice 2 are accurately located, and their spatial range remains consistent across the slice coordinate system and 3D point cloud. This demonstrates that SCTD has high sensitivity and spatial consistency in locating millimeter-scale missing teeth.

For the scratch defects in the groove region, Thread Groove Slices 4, 5, and 6 are selected as representative slices. For each scratch defect, the detection results are sequentially presented with projection visualizations, defect detection results, and point cloud density heatmaps.

**Figure 27:**
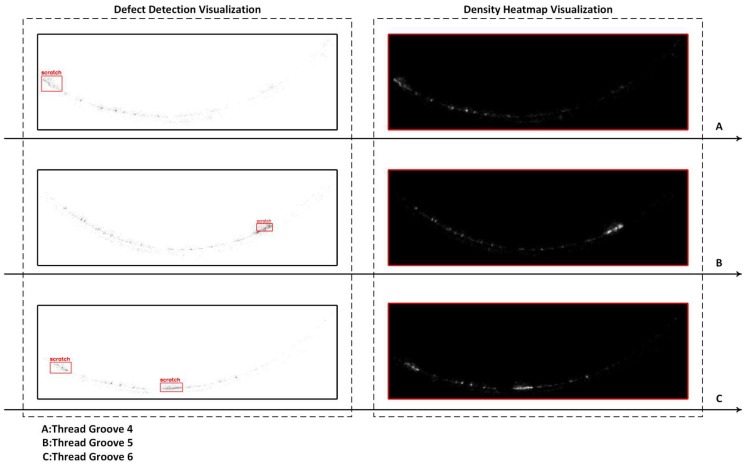
Scratch Detection in Groove Region: Detection results and Point Cloud Density Heatmaps for Groove Slices 4, 5, and 6.

As shown in Figure 27, taking Groove Slice 4 as an example, in the projection view, a long abnormal region extending along the axial direction of the thread can be observed, with the point cloud clearly clustered in the local space. After scratch detection, this region is covered with continuous defect markers in the defect visualization map, with the boundary extending along the groove centerline, and its length is roughly consistent with the actual visible scratch range. The density heatmap further shows that this location forms a high-density band with brightness significantly higher than the surrounding background, confirming the characteristic of the scratch corresponding to the local point cloud anomaly.

In Groove Slices 5 and 6, two more complex scratch types are observed: one is a single long scratch, and the other consists of multiple independent short scratches. In the defect detection results, the corresponding positions are marked, and no false detection occurs between different scratches. The density heatmap shows that these scratch regions form small but consistently strong high-density patches, while scattered noise points in the groove appear as isolated weak bright spots, not misidentified as continuous scratch structures. This demonstrates that LPDE, under multi-scale and spatial continuity constraints, effectively suppresses the false detection risk caused by local high-density noise.

From Figure 25, Figure 26 and Figure 27, SCTD and LPDE are applied to the thread profile and groove regions, respectively, on the same set of 3D point cloud data. Both typical defect types show clear and stable spatial responses: missing teeth form highly consistent local anomalies in curvature peaks, orthogonal residuals, and depth profiles; scratches exhibit multi-view consistent band-like concentration features in projection form, detection markers, and density heatmaps. Compared to methods relying solely on 2D images or single geometric indicators, this 3D detection process demonstrates greater robustness and interpretability in defect location, range, and shape characterization.

After completing the 3D defect detection, this paper further conducts a quantitative evaluation of defect sizes, comparing the actual length, actual depth, and measured values. Table 6 shows the differences between the actual and measured sizes of missing teeth and scratches at different positions. For both missing teeth and scratch defects, the differences between the measured length or depth and the actual values are small, indicating that the system can accurately restore the geometric features of the defects.

The measurement accuracy of the proposed 3D reconstruction framework was evaluated by comparing the measured defect lengths against the ground truth. As summarized in Table 7, Missing Teeth defects have an MAE of 0.083 mm and Scratch defects have an MAE of 0.065 mm, with an overall MAE of 0.074 mm (≈4.1% relative to the average thread pitch), demonstrating millimeter-level accuracy suitable for precision-critical industrial applications.

## 4. Discussion

### 4.1. Performance Evaluation of 3D Detection End Reconstruction Algorithms

This section evaluates three mainstream point cloud reconstruction algorithms: DUST3R [33] (224 × 224 and 512 × 384 models), VGGT [34], and GS. Additionally, the DC-SOR filtering algorithm is used to analyze the optimization effects of different reconstruction algorithms on the original external thread point cloud data. By comparing the point cloud reconstruction results of each algorithm and the improvements after filtering, the advantages and disadvantages of different algorithms in terms of reconstruction quality can be determined.

In this paper, four evaluation metrics—Point Count, Point Cloud Density, Normal Consistency, and Curvature Response—are used to assess the performance of each reconstruction algorithm. The specific calculation formulas are as follows:

(24) PointCount =N

(25) $$\mathrm{Point}\mathrm{Cloud}\mathrm{Density}=\frac{N}{V}$$

(26) $$\mathrm{Normal}\mathrm{Consistency}=\frac{1}{N}\sum_{i=1}^{N}\left(\frac{\left|n_{i}\cdot n_{j}\right|}{\left|n_{i}\right|\left|n_{j}\right|}\right)$$

(27) $$\mathrm{Curvature}\mathrm{Response}=\frac{\sum_{i=1}^{N}\left|\kappa_{i}\right|}{N}$$

N represents the total number of points in the point cloud, which is used to calculate the overall scale of the reconstruction result;

V refers to the 3D spatial volume covered by the point cloud, typically calculated from the bounding box of the point cloud, used to estimate the point cloud density within a unit spatial volume;

$n_{i}$ and $n_{j}$ represent the normal vector of point i and other points within the region, used to calculate the alignment degree of the vectors through spatial geometric relations;

$\kappa_{i}$ is the local curvature of point i; it is used to describe the variation in this point’s local surface curvature, with higher curvature indicating clearer features and more pronounced geometric characteristics.

**Figure 28:**
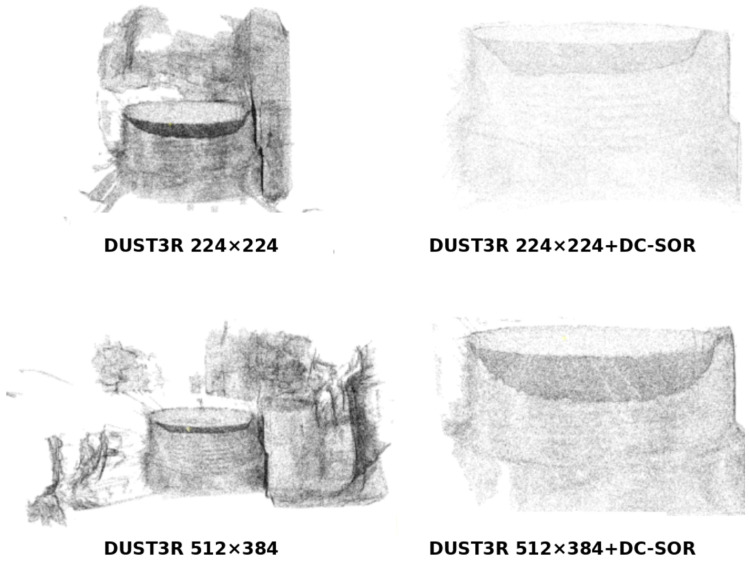
Point Cloud Reconstruction Results of DUST3R Models (224 × 224 and 512 × 384) with and without DC-SOR Filtering.

Combining the visualization results in Figure 28 with the quantitative data in Table 8, both DUST3R models exhibit clear point cloud sparsity prior to reconstruction: the Point Count is only 1.18 × 10^5^ and 1.53 × 10^5^, and the Point Cloud Density is 65.59 and 93.53, respectively. The reconstructed external thread profile is discontinuous, with a large number of points scattered across the background and support structure. After DC-SOR filtering, the point count of both DUST3R models was reduced to approximately 40% of the original (for example, the 224 × 224 model decreased from 117,851 to 45,362). Normal Consistency and Curvature Response showed only slight improvements (Normal Consistency increased by about 0.02, and Curvature Response improved by about 0.04–0.05), but a clear thread tooth hierarchy was still difficult to form in the image. This indicates that, under the condition of the point cloud being too sparse, filtering can only serve a limited “background cleaning” function and cannot fundamentally improve the 3D reconstruction quality of the external thread. Therefore, DUST3R is more suitable as a coarse geometric recovery method and is not applicable to external thread industrial detection scenarios with millimeter-scale defects.

**Figure 29:**
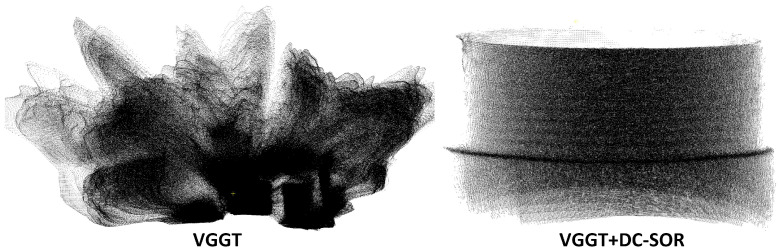
Point Cloud Reconstruction Results of VGGT with and without DC-SOR Filtering.

VGGT, when unfiltered, can generate a dense point cloud with a count close to 1.0 × 10^6^ and a Point Cloud Density of 193.86, with an overall smooth surface. However, as shown in Figure 29, a large number of points are concentrated in the burr-like background regions outside the workpiece. After DC-SOR filtering, the point count of VGGT drastically decreases to 192,454, with the background point cloud significantly suppressed. Normal Consistency improves from 0.6973 to 1.3090, and Curvature Response increases from 0.1994 to 0.3378, indicating that the filtering effectively removed normal disruptions and high-noise regions. However, from the visualization results, the filtered VGGT appears more like a smooth cylinder, with the fine undulations of the thread crest and trough significantly weakened. Local curvature variations are flattened, making it more suitable for modeling large scenes or flat surfaces, but it is challenging to meet the defect detection requirements for external threads, which are high-frequency geometric structures.

**Figure 30:**
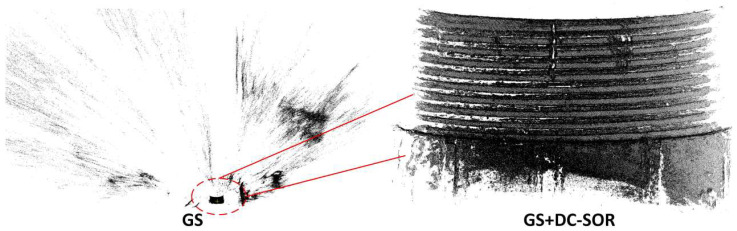
Point Cloud Reconstruction Results of GS with and without DC-SOR Filtering.

As shown in Figure 30, the original reconstruction of GS results in an extremely large point cloud set, which contains a significant amount of irrelevant background information. The high sensitivity brought by GS’s adaptive densification advantage leads to ‘anomalies’ in the original evaluation metrics: the original GS point cloud has a Point Count of 1.52 × 10^6^, but a Point Cloud Density of only 105.16, Normal Consistency of 0.3397, and Curvature Response of −0.6726, reflecting the significant downward impact of noise and background points on the overall statistics. In contrast, GS + DC-SOR is able to provide the most detailed thread structure reconstruction.

After introducing the DC-SOR filtering, the point count of GS is reduced from 1,521,202 to 246,469, but the Point Cloud Density jumps from 105.16 to 301.69, indicating that a large amount of redundant background points are removed, and the points in the thread region are further concentrated. At the same time, Normal Consistency increases from 0.3397 to 1.2795, and Curvature Response improves from −0.6726 to 0.2986, with normal and curvature features transitioning from being ‘drowned by noise’ to ‘highlighting the thread structure’.

**Figure 31:**
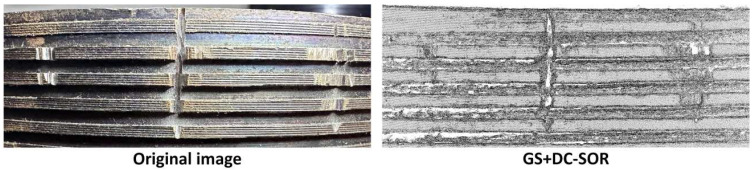
Comparison of Original Image and GS + DC-SOR Optimized Point Cloud.

We enlarged the optimized point cloud of GS + DC-SOR and compared it with the original defect image. As shown in Figure 31, the GS + DC-SOR strategy optimizes the point cloud data, accurately restoring the external thread defects. The filtered GS not only preserves the multi-layered tooth profile and trough details of the thread but also forms significant depth and curvature changes in the defect areas, providing highly recognizable geometric features for subsequent SCTD and LPDE detection.

It is worth emphasizing that, numerically, VGGT + DC-SOR is slightly higher than GS + DC-SOR in Normal Consistency (1.3090 vs. 1.2795) and Curvature Response (0.3378 vs. 0.2986), but from the visualization results, it can be seen that, under similar evaluation metrics, the two methods exhibit completely different geometric properties: VGGT + DC-SOR tends to generate a “highly smooth but almost featureless cylindrical surface,” while GS + DC-SOR, while smooth overall, retains fine-grained curvature changes at the thread crest, trough, and defect grooves, making the depth and curvature responses of missing teeth and scratch highly prominent in point cloud slices. Therefore, if global normal and curvature statistics are used as the sole basis for judgment, VGGT’s suitability for industrial detection may be overestimated, neglecting its excessive smoothing of high-frequency details. A comprehensive analysis of the visual results and the data in Table 7 shows that GS + DC-SOR achieves a better balance of “background suppression + detail preservation” while maintaining excellent evaluation metrics, making it more suited to the comprehensive requirements of external thread defect detection for point cloud density, local geometric sensitivity, and interpretability.

In conclusion, DUST3R is limited by its inherent point cloud sparsity in external thread scenarios, and DC-SOR can only improve its reconstruction quality to a limited extent. VGGT, although it provides high-density, uniform point clouds and good global metrics, results in overly smooth reconstructions that are not conducive to the expression of millimeter-scale defects. In contrast, GS combined with DC-SOR not only significantly improves normal consistency and curvature response, but also further compresses redundant background points, increases the point cloud density in target areas, and preserves clear geometric layers and depth variations in the thread profile and defect areas. Therefore, GS + DC-SOR is considered the most suitable 3D reconstruction and filtering combination for this system, exhibiting the best overall performance and engineering application potential in external thread 3D detection.

### 4.2. System Security Evaluation

In this study, the security defense mechanism mainly focuses on evaluating the robustness of the proposed detection framework under abnormal image perturbations. Specifically, alpha-channel attack experiments are introduced to analyze the influence of image disturbances on both the 2D detection module and the 3D reconstruction process.

This section verifies the system’s vulnerability by simulating an alpha-channel image attack [35]. The attack involves overlaying an adversarial patch onto the image’s alpha channel, causing subtle pixel-level disturbances that are nearly imperceptible to the human eye but significantly affect the depth models, thus disrupting the detection system.

**Figure 32:**
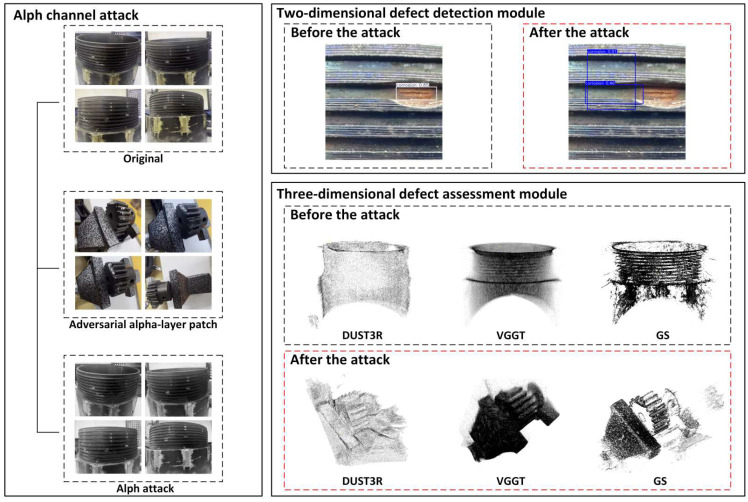
Comparison of Alpha Channel Attack on 2D and 3D Detection Modules (Before and After Attack).

As shown in Figure 32, in the 2D defect detection module, YOLOv13 accurately detects defects before the attack, with defect locations highly consistent with manual annotations. After the attack, the detection boxes fail to cover the correct defect areas, leading to missed detections and false positives. This failure introduces two major risks: one is that defective parts may be misjudged as qualified and enter downstream processes; the other is that a large number of qualified parts may be misjudged as defective, causing production delays and resource waste.

These experiments highlight the destructive impact of alpha-channel attacks, showing that even minor and nearly undetectable changes to image data can trigger a complete failure of the entire detection chain. Such attacks lead to significant failures in both 2D and 3D detection, exposing the inadequacy of traditional models that evaluate accuracy only under “benign data” conditions.

To mitigate this, the system must incorporate data integrity verification and access control mechanisms to prevent malicious tampering of images before they enter the detection modules. Additionally, anomaly detection and cross-modal consistency checks should be designed within the 2D and 3D modules to ensure global geometric consistency of the reconstructed point cloud and prevent detection results from significantly deviating from historical statistical patterns. Only by combining strong algorithmic performance with robust security measures can the system ensure stable and reliable operation in complex industrial environments, meeting the credibility requirements for engineering applications.

To mitigate the influence of alpha-channel attacks, the proposed system adopts a channel consistency preprocessing strategy before both 2D detection and 3D reconstruction. Specifically, all input images are forcibly converted into standard RGB three-channel images, and any additional alpha-channel information is removed during preprocessing.

Since the proposed framework only relies on RGB visual information for defect analysis and reconstruction, eliminating the alpha channel can effectively suppress hidden perturbations introduced through transparency manipulation while maintaining the original image content. This lightweight preprocessing strategy improves the robustness and stability of the system under abnormal image inputs.

## 5. Conclusions

This paper proposes a multi-dimensional vision-based external thread defect detection system integrating 2D detection, 3D reconstruction, and robustness-oriented analysis. The proposed framework addresses the limitations of conventional 2D inspection methods in geometric characterization by combining image-based defect localization with point cloud-based quantitative analysis. Through in-depth research on vision-based external thread defect detection, the following conclusions are drawn:

1. Custom External Thread Image Acquisition System: A dedicated external thread image acquisition system is designed to provide stable multi-view image inputs for both 2D defect detection and 3D reconstruction. The controlled lighting and motion mechanisms improve image consistency and provide reliable data support for subsequent analysis.
2. Multi-Dimensional Defect Detection Framework: A multi-dimensional external thread defect detection framework integrating 2D detection and 3D reconstruction is proposed. The YOLOv13-based detection module achieves reliable detection performance for missing teeth, scratches, and corrosion defects under complex industrial backgrounds. Furthermore, the Gaussian Splatting-based reconstruction method successfully recovers detailed geometric structures of external threads from multi-view images, enabling point cloud-based quantitative analysis.
3. Point Cloud-Based Defect Quantification Method: To improve the quality of reconstructed point clouds, a dual-constrained statistical outlier removal (DC-SOR) strategy is introduced to suppress noise while preserving thread boundary structures. Based on the optimized point cloud, the proposed Slice Curvature Tooth Defect (SCTD) and Local Point Density Estimation (LPDE) methods achieve effective geometric characterization and millimeter-level defect size estimation for missing teeth and scratch defects.
4. Robustness Analysis under Image Perturbations: This paper further analyzes the influence of image perturbations on vision-based industrial inspection systems. Experimental results demonstrate that alpha-channel disturbances may affect both defect detection and 3D reconstruction performance.

The Corrosion class achieves a lower AP@0.5 (0.797) than Missing Tooth (0.953) and Scratch (0.967) due to irregular morphology, weak texture boundaries, and higher intra-class variability under complex illumination. While YOLOv13n performs well overall, future work could explore incorporating color-space features, multi-spectral imaging, or attention-based feature fusion to further improve corrosion detection.

## Figures and Tables

**Table 1:** Generative Models and Their Evolution: GAN, StyleGAN, SAN, and StyleSAN-XL.

Model	Core Concept	Innovation and Optimization	Evolutionary Relationship
**GAN**	Generator and discriminator compete to generate data.	Learns adversarially to generate data.	Foundation of generative models.
**StyleGAN**	GAN with a style module to enhance images.	Controls image generation via style-based learning.	Enhances GAN for better image quality.
**SAN**	Optimizes GAN’s discriminator.	Uses optimal transport for training stability.	Improves GAN’s training stability.
**StyleSAN-XL**	Combines StyleGAN and SAN for large-scale generation.	Merges style control with SAN’s optimization for better quality.	Combines StyleGAN and SAN to improve quality and stability.

**Table 2:** Contributions of YOLOv13’s Innovative Modules to System Adaptability.

Module	Function	Contribution
**Depthwise Separable Convolution**	Reduces computation, boosts inference speed	Fast extraction of small defect features, ensuring real-time precision
**Hypergraph Convolution**	Models context relationships between targets, reduces background noise	Accurate defect localization reduces interference from complex backgrounds
**Adaptive Multi-Scale Feature Fusion**	Combines features from different scales, enhances defect perception	Stable detection of defects from small scratches to large missing teeth, balancing precision and recall

**Table 3:** Results of training data for different defect detection.

Class	Precision	Recall	F1-Score	AP@0.5
Missing Tooth	0.91	0.96	0.93	0.953
Scratch	0.92	0.98	0.95	0.967
Corrosion	0.84	0.91	0.87	0.797
Overall	0.89	0.97	0.88	0.906

**Table 4:** Functional Contributions of YOLOv13 Modules to Detection Adaptability.

Model	mAP	Latency (ms)	Model Size (MB)	GFLOPs	Params (M)
YOLOv5n	86.4	3.1	10.0	7.1	2.5
YOLOv6n	88.1	3.8	16.8	11.8	4.2
YOLOv8n	89.3	3.3	12.0	8.2	3.0
YOLOv9t	90.1	3.0	**8.4**	8.5	**2.1**
YOLOv11n	90.2	2.7	10.0	6.3	2.5
YOLOv12n	90.4	2.8	10.5	6.5	2.6
YOLOv13n	**90.6**	**2.6**	9.8	**6.2**	2.4

**Table 5:** Ablation Study on the Ratio of Synthetic Images in the Training Dataset.

Setting	Real Images	Synthetic Images	Total Images	Precision	Recall	mAP@0.5
S0	600	0	600	0.842	0.801	0.817
S1	600	600	1200	0.865	0.830	0.840
S2	600	3000	3600	0.876	0.855	0.855
S3	600	6000	6600	0.884	0.910	0.875
S4	600	15,000	15,600	0.89	0.97	0.906

**Table 6:** Comparison of Actual and Measured Sizes for Missing Teeth and Scratch Defects at Different Positions.

Defect Type	Defect Position	Actual Length (mm)	Measured Length (mm)	Actual Depth (mm)	Measured Depth (mm)
Missing teeth	A	1.51	1.43	1.23	1.19
	B	1.83	1.75	1.37	1.44
	C	1.58	1.46	1.05	0.97
	D	1.19	1.14	0.87	0.85
Scratch	A	1.87	1.82	---	---
	B	2.52	2.41	---	---
	C	3.06	3.11	---	---
	D	1.76	1.81	---	---

**Table 7:** Measurement Errors of Reconstructed External Thread Defects.

Defect Type	MAE (mm)	Std Dev (mm)	% Error (Relative to Pitch)
Missing Teeth	0.083	0.028	5.4%
Scratch	0.065	0.026	2.8%
Overall	0.074	0.027	4.1%

**Table 8:** Performance Evaluation of 3D Reconstruction Algorithms (DUST3R, VGGT, GS) with and without DC-SOR Filtering.

Model	Point Count	Point Cloud Density	Normal Consistency	Curvature Response
DUST3R 224 × 224	117,851	65.5891	1.3351	0.2116
DUST3R 224 × 224 +DC-SOR	45,362	61.1345	1.3550	0.2530
DUST3R 512 × 384	152,550	93.5301	1.3741	0.1873
DUST3R 512 × 384 +DC-SOR	51,330	89.2154	1.3945	0.2390
VGGT	993,876	193.8614	0.6973	0.1994
VGGT +DC-SOR	192,454	172.2973	1.3090	0.3378
GS	1,521,202	105.1556	0.3397	−0.6726
GS +DC-SOR	246,469	301.6895	1.2795	0.2986

## Data Availability

Due to industrial confidentiality and equipment-related restrictions, part of the self-collected defect image data and reconstructed point cloud data are not publicly available. Processed data supporting the findings of this study are available from the corresponding author upon reasonable request.

## Abbreviations

GAN	Generative Adversarial Network
StyleGAN	Style Generative Adversarial Network
SAN	Style-based Adversarial Network
StyleSAN-XL	Style-based Self-Attention Network with Extended Large-scale Generation
YOLOv13	You Only Look Once version 13
DC-SOR	Dual-Constrained Statistical Outlier Removal
SCTD	Slice Curvature-based Tooth-Missing Detection
LPDE	Local Point Density Estimation
GS	Gaussian Splatting
FID	Fréchet Inception Distance
PCA	Principal Component Analysis
DBSCAN	Density-Based Spatial Clustering of Applications with Noise
VGGT	Visual Geometry Grounded Transformer
DUST3R	Deep Unsupervised Spatial-Temporal Reconstruction
